# Quantitative CT assessment of intrathoracic visceral fat depots and their association with incident heart failure in asymptomatic adults

**DOI:** 10.3389/fcvm.2026.1820542

**Published:** 2026-05-28

**Authors:** Cheng-Chun Yang, Chung-Lieh Hung, Chen-Yen Chien, Yang-Wen Hsieh, Wei-Ming Huang, Chun-Ho Yun, Ricardo C. Cury

**Affiliations:** 1 Division of Cardiothoracic Radiology, Department of Medical Imaging, Chi Mei Medical Center, Tainan, Taiwan; 2 Institute of Biomedical Sciences, MacKay Medical College, New Taipei, Taiwan; 3 Division of Cardiology, Department of Internal Medicine, MacKay Memorial Hospital, Taipei, Taiwan; 4 Division of Cardiovascular Surgery, Department of Surgery, MacKay Memorial Hospital, Taipei, Taiwan; 5 Department of Medical Research, MacKay Memorial Hospital, Taipei, Taiwan; 6 Department of Radiology, MacKay Memorial Hospital, Taipei, Taiwan; 7 Department of Medicine, MacKay Medical College, New Taipei, Taiwan; 8 MacKay Junior College of Medicine, Nursing, and Management, New Taipei, Taiwan; 9 Department of Radiology, Florida International University, Baptist Health of South Florida, Miami, FL, United States

**Keywords:** cardiovascular diseases, ectopic fat depots, epicardial adipose tissue, heart failure, obesity

## Abstract

**Background:**

Epicardial adipose tissue (EAT) is a well-recognized cardiometabolic risk factor. However, the prognostic significance of other intrathoracic visceral adipose tissue (VAT) in heart failure (HF) development and phenotypes remains incompletely understood. This study examined the associations between distinct intrathoracic VAT depots and incident HF in asymptomatic adults.

**Methods:**

We retrospectively analyzed 3,094 asymptomatic adults (mean age: 49.7 ± 9.7 years) undergoing cardiac CT for health screening. Baseline volumes of intrathoracic VAT depots, including EAT, peri-aortic root fat (PARF), and thoracic peri-aortic adipose tissue (TAT), were quantified using a semi-automated method. Left ventricular mass index (LVMI) was assessed by echocardiography. Associations between intrathoracic VAT and LVMI were evaluated using restricted cubic spline regression. Incident HF was analyzed using Cox regression models and Kaplan–Meier survival curves.

**Results:**

During a median follow-up of 10.3 years, 140 participants developed HF, of whom 89.9% had preserved ejection fraction (HFpEF). Men demonstrated greater intrathoracic VAT accumulation with increasing BMI (*p*-interaction <0.05). EAT increased more steeply with rising BMI among those who developed HF (*p*-interaction = 0.011) and showed the strongest association with LVMI. Higher tertiles of intrathoracic VAT volumes and BMI were associated with increased HF incidence (log-rank *p* < 0.001). After multivariable adjustment, both EAT and PARF remained independently associated with incident HF (hazard ratio per 1-unit *Z*-score increase: 1.23 [95% CI: 1.04–1.46] and 1.31 [95% CI: 1.09–1.57], respectively), with optimal cutoffs estimated to be 71.37 cm^3^ and 23.50 cm^3^, respectively.

**Conclusion:**

EAT and PARF are independently associated with incident HF, predominantly HFpEF, and may serve as novel imaging biomarkers for early risk stratification.

## 1 Introduction

Heart failure (HF) is a heterogeneous clinical syndrome that imposes a substantial global health and economic burden, affecting approximately 64 million individuals worldwide (1). About half of all HF cases are classified as heart failure with preserved ejection fraction (HFpEF), a phenotype with a steadily rising prevalence—likely driven by an aging population and the increasing rates of comorbidities such as metabolic syndrome and diabetes mellitus (DM), both closely associated with excess body fat (2, 3).

Obesity is a major modifiable risk factor for HFpEF, with over 80% of cases occurring in individuals who are overweight or obese (4, 5). Rather than being merely a comorbidity, obesity is increasingly recognized as a direct contributor to HFpEF development through its adverse impact on myocardial structure, function, and metabolism (3, 6, 7). Beyond overall adiposity, emerging evidence indicates that intrathoracic visceral adipose tissue (VAT) may play a critical role in HFpEF pathogenesis (8, 9). In particular, epicardial adipose tissue (EAT), situated within the pericardial sac, has drawn significant attention due to its anatomical proximity to and shared microcirculation with the myocardium (10). Large cohort studies, including the Framingham Heart Study and the Multi-Ethnic Study of Atherosclerosis (MESA), have linked higher EAT volume to heightened risks of cardiovascular diseases (CVD) and adverse left ventricular remodeling (11–15). Additional associations with hypertension, DM, and metabolic syndrome further support its contribution to HF development (16–19). Other intrathoracic VAT depots, such as peri-aortic root fat (PARF) and thoracic peri-aortic adipose tissue (TAT), have also been linked to metabolic and cardiovascular risk, yet their associations with HF remain poorly defined (20–23). These VAT depots are clinically relevant and amenable to reliable quantification using non-invasive imaging modalities.

Recent advances in multi-detector computed tomography (MDCT) and postprocessing techniques have expanded the diagnostic utility of cardiac CT beyond coronary artery assessment, enabling reproducible quantification of various cardiac and pericardial structures, including intrathoracic VAT depots (24). Accordingly, this study aimed to quantify the volumes of EAT, PARF, and TAT using cardiac CT, and to evaluate their associations with incident HF in asymptomatic adults.

## 2 Methods

### 2.1 Study population

Between January 2005 and December 2012, 3,411 consecutive participants who underwent non-contrast ECG-gated cardiac CT for coronary artery calcium (CAC) scoring as part of a health examination at a tertiary medical center were enrolled. None of the participants reported any clinical symptoms such as chest pain, angina, or dyspnea. Comprehensive physical examinations were conducted, and baseline characteristics and medical histories—including CVD, DM, hypertension, smoking, and physical activity levels—were collected via structured questionnaires. CVD history encompassed prior myocardial infarction, coronary artery disease, hospitalizations for congestive HF, stroke, or peripheral arterial disease. Participants with a known history of HF were excluded. Hypertension was defined as two or more blood pressure measurements >140/90 mmHg or current use of antihypertensive medications. DM was diagnosed based on fasting glucose levels ≥126 mg/dL on two separate tests or ongoing treatment with diabetic medications. The study was approved by the local ethics committee and conducted in accordance with the Declaration of Helsinki.

### 2.2 Multi-detector computed tomography (MDCT) scanning protocol

The scan utilized a 16-slice MDCT scanner (Sensation 16, Siemens Medical Solutions, Forchheim, Germany), configured with 16 mm × 0.75 mm collimation, a tube voltage of 120 kV, and a rotation time of 420 ms. Image acquisition took place during a single breath-hold, extending from above the tracheal bifurcation to just below the heart apex, using prospective ECG triggering centered at 70% of the R-R interval. The raw data were reconstructed into 3 mm thick axial slices with no overlap, using a standard kernel and a 25 cm field of view.

### 2.3 Measurement of intrathoracic VAT depots

**Figure 1:**
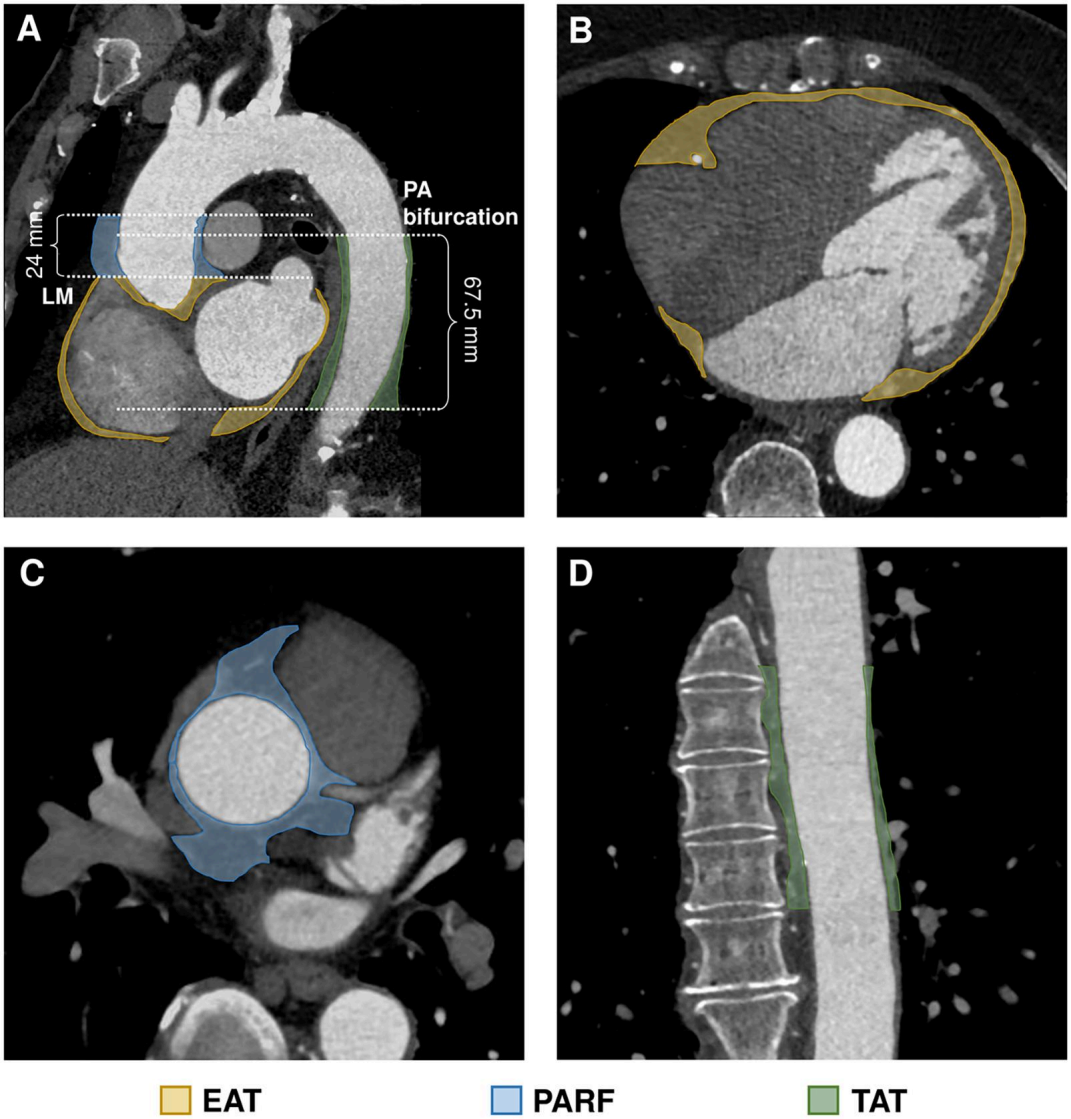
Measurement of intrathoracic visceral adiposity. Annotated CT images showing segmentation and quantification of epicardial adipose tissue [EAT; **(B)**], peri-aortic root fat [PARF; **(C)**], and thoracic peri-aortic adipose tissue [TAT; **(D)**]. Key landmarks include the pulmonary artery (PA) bifurcation and left main (LM) coronary artery **(A)**.

EAT, PARF, and TAT were all quantified from CT images using a dedicated workstation (Aquarius 3D Workstation, TeraRecon, San Mateo, CA, USA). All measurements were performed independently by two experienced cardiothoracic radiologists. A semi-automatic segmentation technique, developed for quantifying regional fat volumes, was used across all fat depots. Fat tissue was identified as pixels within a range of −195 to −45 Hounsfield Units (HU), with a window center at −120 HU. EAT was defined as any adipose tissue located within the pericardial sac (Figures 1A,B). PARF was delineated by manually tracing the pericardium on eight consecutive axial slices, extending 24 mm cranially from the level of the left main coronary artery, to yield a cap of fat surrounding the aortic root and overlying the EAT (Figures 1A,C), as described in our previous work (21). TAT was defined as adipose tissue surrounding the thoracic descending aorta, measured over a 67.5 mm segment caudal to the level of the pulmonary artery bifurcation (Figures 1A,D). This specific segment, consistent with the definition used in the Framingham Heart Study, was chosen to standardize the measurement across individuals and to minimize variability related to thoracic aortic length, compared to using total TAT volume (25). The volume of each fat compartment was calculated by summing all fat voxels, followed by 3D reconstruction. The reliability of fat measurements was validated by performing repeated analyses on 50 randomly selected cases, with the evaluators blinded to the original measurements and clinical information. The Bland–Altman method was applied to assess the 95% limits of agreement and calculate the coefficient of variation (COV). The intra-observer COVs for EAT, PARF, and TAT measurements were 4.27%, 4.87%, and 4.82%, respectively, while the inter-observer COVs were 5.41%, 6.58%, and 6.81%, respectively.

### 2.4 Left ventricular measurements

Echocardiographic assessments were conducted to evaluate several anatomical parameters of the left ventricle (LV), including septal wall thickness (mm), posterior wall thickness (mm), internal diameter (mm), LV mass (g), and LV mass index (LVMI) (g/m^2^). The LVMI was calculated using the formula: LVMI = LV mass/body surface area (BSA). LV hypertrophy was defined as an LVMI >115 g/m^2^ for men and >95 g/m^2^ for women. The relative wall thickness (RWT) was calculated using the formula: RWT = (2 × posterior wall thickness)/LV internal diameter at end-diastole. LV geometric patterns were further classified on the basis of LVMI and RWT as concentric remodeling (normal LVMI and increased RWT), eccentric hypertrophy (increased LVMI and normal RWT), concentric hypertrophy (increased LVMI and increased RWT), or normal geometry (normal LVMI and normal RWT) (26). Baseline left ventricular ejection fraction (LVEF) was measured in all asymptomatic participants using the 2D-guided linear or Biplane Simpson's method, in accordance with contemporary guidelines (26). In participants who subsequently developed HF, follow-up LVEF assessment and classification of clinical HF phenotypes were performed using the same method, with HFpEF defined as LVEF ≥ 50%. Additionally, LV hypertrophy and geometric patterns, based on relative wall thickness, were analyzed and categorized as concentric remodeling, eccentric hypertrophy, concentric hypertrophy, or normal geometry.

### 2.5 Study outcome

The primary study endpoint was incident HF, defined as a new clinical diagnosis of HF during the study period. Incident HF was determined based on the first hospital admission for HF with characteristic symptoms and/or signs (e.g., breathlessness, fatigue, pulmonary congestion/edema, or ankle swelling) and objective evidence of elevated brain natriuretic peptide (BNP, ≥100 pg/mL) or N-terminal prohormone of BNP (NT-proBNP, ≥300 pg/mL), after excluding non-cardiac causes (27). All HF admissions were adjudicated by two experienced cardiologists.

### 2.6 Statistical analysis

Continuous variables were expressed as mean ± standard deviation (SD), while categorical variables were presented as frequencies and percentages. Baseline demographic and anthropometric differences between groups were evaluated using analysis of variance (ANOVA) for continuous variables and chi-square or Fisher's exact tests for categorical variables, as appropriate. Restricted cubic spline regression was employed to examine the relationships between intrathoracic VAT and BMI with LVMI. Multivariable Cox proportional hazards models were used to assess the independent associations between intrathoracic VAT depots and incident HF. Kaplan–Meier survival analysis was conducted to evaluate cumulative HF incidence across tertiles of VAT depots volumes and BMI, with comparisons performed using the log-rank test. Statistical significance was determined at a two-tailed *p*-value of <0.05. All statistical analyses were conducted using SPSS (IBM Corp., Armonk, NY), Stata version 14 (Stata Corp., College Station, TX) and SAS version 9.2 (SAS Institute, Cary, North Carolina).

## 3 Results

### 3.1 Baseline characteristics of all study participants

A total of 3,094 participants (mean age: 49.7 ± 9.7 years; 72.4% male) met the inclusion criteria out of 3,411 initially screened. Exclusions comprised participants with missing baseline data, such as anthropometric measurements and medical history (*n* = 293), and those for whom intrathoracic VAT measurements could not be performed (*n* = 24), as the data could not be processed by the software (Supplementary Figure S1). The baseline demographic characteristics of the included participants are summarized in Table 1. The baseline BMI and volumes of EAT, PARF, and TAT were 24.7 ± 3.5 kg/m^2^, 75.9 ± 30.6 cm^3^, 21.6 ± 11.4 cm^3^, and 7.1 ± 3.9 cm^3^, respectively.

**Table 1:** Baseline demographics.

	Non-HF (*n* = 2,954)	HF (*n* = 140)	All (*n* = 3,094)	*p*-value
Age (years)	49.2 ± 9.4	59.3 ± 11.3	49.7 ± 9.7	<0.001
SBP (mmHg)	122.7 ± 16.7	129.4 ± 19.1	123.0 ± 16.9	<0.001
DBP (mmHg)	76.2 ± 10.9	77.6 ± 10.9	76.2 ± 10.9	0.142
Pulse rate (bpm)	73.3 ± 9.8	73.5 ± 10.3	73.4 ± 9.8	0.854
Body fat composition (%)	25.92 ± 6.48	29.43 ± 8.13	26.08 ± 6.60	<0.001
Sex				
Male, *n* (%)	2,147 (72.7%)	92 (65.7%)	2,239 (72.4%)	0.072
Female, *n* (%)	807 (27.3%)	48 (34.3%)	855 (27.6%)	
Anthropometric measurement				
Weight (kg)	68.47 ± 12.42	69.84 ± 12.83	68.54 ± 12.44	0.206
WC (cm)	83.45 ± 12.09	86.02 ± 19.87	83.56 ± 12.54	0.021
BC (cm)	94.18 ± 10.45	94.46 ± 18.49	94.19 ± 10.92	0.772
Medical history				
Hypertension, *n* (%)	450 (15.2%)	60 (42.9%)	510 (16.5%)	<0.001
Hyperlipidemia, *n* (%)	144 (4.9%)	14 (10.0%)	158 (5.1%)	0.007
CVD, *n* (%)	108 (3.7%)	26 (18.6%)	134 (4.3%)	<0.001
Stroke, *n* (%)	12 (0.4%)	1 (0.7%)	13 (0.4%)	0.059
Diabetes, *n* (%)	150 (5.0%)	25 (18.0%)	175 (5.6%)	<0.001
Smoking, *n* (%)	341 (11.5%)	23 (16.5%)	360 (11.6%)	0.070
myocardial infarction, *n* (%)	9 (0.3%)	1 (0.7%)	10 (0.3%)	0.404
Lab tests				
Cholesterol (mg/dL)	201.72 ± 35.43	207.10 ± 55.62	201.97 ± 36.60	0.091
LDL (mg/dL)	130.50 ± 32.15	128.15 ± 35.55	130.40 ± 32.31	0.402
HDL (mg/dL)	52.38 ± 14.15	51.94 ± 13.69	52.36 ± 14.12	0.725
Triglyceride (mg/dL)	140.19 ± 94.01	174.53 ± 331.39	141.75 ± 115.99	<0.001
HbA1c (%)	5.84 ± 0.85	6.36 ± 1.27	5.87 ± 0.89	<0.001
HOMA-IR index	2.00 ± 1.56	2.25 ± 1.53	2.02 ± 1.56	0.230
NT-proBNP (pg/dL)	33.99 ± 33.87	153.30 ± 422.08	40.58 ± 107.59	<0.001
Hs-CRP (mg/L)	0.20 ± 0.42	0.27 ± 0.31	0.20 ± 0.42	0.137
eGFR (mL/min)	83.68 ± 15.97	76.14 ± 21.32	83.34 ± 16.33	0.001

BC, buttock circumference; CRP, c-reactive protein; CVD, cardiovascular disease; DBP, diastolic blood pressure; eGFR, estimated glomerular filtration rate; HDL, high-density lipoprotein; HbA1c, glycohemoglobin; HOMA-IR index, homeostatic model assessment of insulin resistance; hs-CRP, high-sensitivity c-reactive protein; LDL, low-density lipoprotein; NT-proBNP, N-terminal pro B-type natriuretic peptide; SBP, systolic blood pressure; WC, waist circumference.

Data are presented as mean ± SD unless otherwise specified.

### 3.2 Sex differences in intrathoracic VAT accumulation

**Figure 2:**
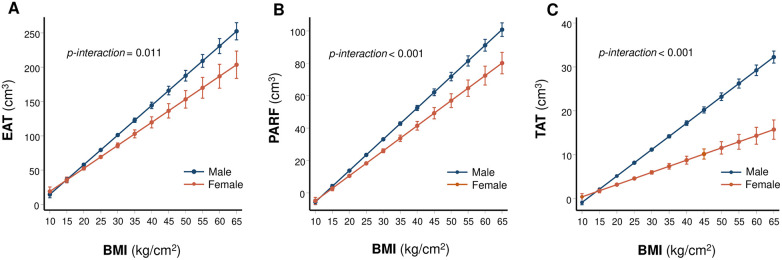
Association between BMI and intrathoracic visceral adiposity stratified by Sex. Caption: Line plots depicting the relationship between body mass index (BMI) and intrathoracic adiposity—epicardial adipose tissue [EAT; **(A)**], peri-aortic root fat [PARF; **(B)**], and thoracic peri-aortic adipose tissue [TAT; **(C)**]—for males (blue) and females (orange), with age adjustment. Error bars represent the standard errors of the mean.

In sex-stratified analyses adjusted for age, significant sex interactions in the associations between BMI and all intrathoracic VAT types were observed (EAT: *p*-interaction = 0.011; PARF and TAT: *p*-interaction <0.011). Males exhibited steeper increases in VAT depot volumes with rising BMI compared to females, with the sex disparity becoming more pronounced at higher BMI levels (Figure 2).

### 3.3 Association of intrathoracic VAT and BMI with LVMI

**Figure 3:**
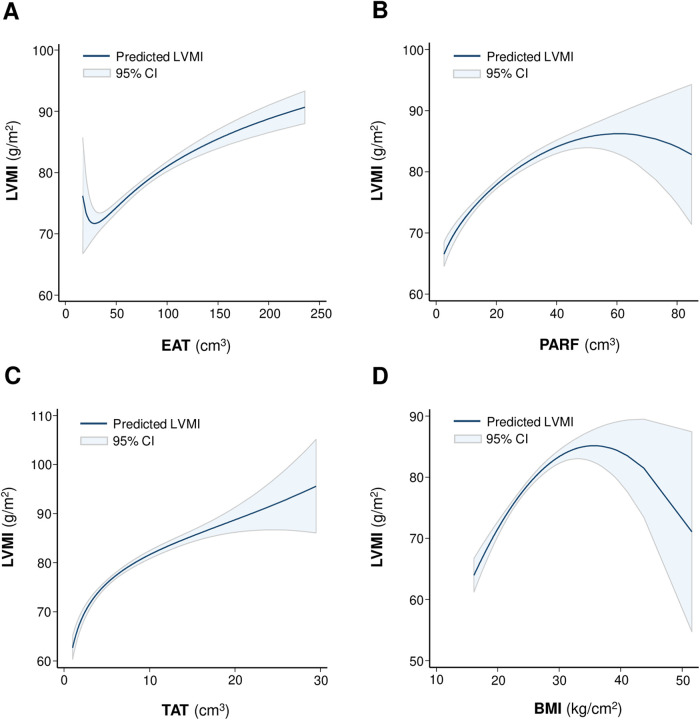
Association of intrathoracic visceral adiposity and BMI with LVMI. Caption: Restricted cubic spline plots depicting the relationship between predicted left ventricular mass index (LVMI; blue lines) and epicardial adipose tissue [EAT; **(A)**], peri-aortic root fat [PARF; **(B)**], thoracic peri-aortic adipose tissue [TAT; **(C)**], and body mass index [BMI; **(D)**], with shaded areas indicating 95% confidence intervals.

In restricted cubic spline analysis, EAT exhibited a steady and strong positive association with LVMI (Figure 3A). In contrast, both PARF and TAT demonstrated non-linear relationships with LVMI. LVMI increased with rising PARF volumes but plateaued at higher levels (Figure 3B). Although TAT showed a positive association with LVMI across the entire range, it exhibited greater variability at higher levels (Figure 3C). Collectively, these findings underscore the positive correlation between intrathoracic VAT and LVMI, with EAT showing the strongest association. Interestingly, BMI also displayed a non-linear relationship with LVMI, where LVMI increased in the non-obese range (BMI 20–30) but decreased in the obese range (BMI > 30) (Figure 3D).

### 3.4 Comparison of baseline characteristics between participants with and without HF development

Over a median follow-up of 10.3 years (interquartile range [IQR]: 4.0–11.0), 140 participants developed new-onset HF, including 48 women and 92 men (cumulative incidence: 5.61% vs. 4.11%; overall 4.53%), and were categorized as the HF group. The remaining 2,954 participants who remained HF-free were categorized as the non-HF group. Among HF cases with available ejection fraction (EF) data, 107 (89.9%) had preserved LVEF (≥50%), whereas 12 (10.1%) had reduced LVEF (<50%). During follow-up, 242 participants died (81 from cardiovascular causes and 161 from non-cardiovascular causes) before a documented HF event and were treated as censored observations in the time-to-event analysis. Among the 81 cardiovascular deaths, heart failure with reduced ejection fraction (HFrEF) was identified in 57 cases only at the final diagnosis at death, without prior documented clinical HF during follow-up; these cases were therefore not classified as incident HF events. A comparison of baseline characteristics and clinical features at the time of HF onset between HFpEF and HFrEF patients within the HF group is provided in Supplementary Table S1.

At baseline, the HF group was significantly older (59.14 ± 11.4 years vs. 49.2 ± 9.4 years, *p* < 0.001) and exhibited higher body fat composition (*p* < 0.001), elevated systolic blood pressure (*p* < 0.001), and larger waist circumference (*p* = 0.021). The HF group also had a higher prevalence of hypertension, CVD, DM, and hyperlipidemia (all *p* < 0.001). Furthermore, the HF group exhibited a less favorable biochemical profile, including elevated levels of triglycerides and NT-proBNP, along with lower estimated glomerular filtration rate (eGFR) (all *p* < 0.001). Echocardiographic findings revealed increased LV septal and posterior wall thickness, larger LV internal diameter, and higher LV mass and LVMI in the HF group (all *p* < 0.001). Although LV hypertrophy prevalence was not statistically significant (*p* = 0.057), the HF group more frequently exhibited abnormal LV geometry, with a greater proportion showing abnormal LV geometry (*p* = 0.003) (Table 2).

**Table 2:** Measurements of intrathoracic visceral adiposity and left ventricle.

	Non-HF (*n* = 2,954)	HF (*n* = 140)	Total (*n* = 3,094)	*p*-value
CT (*n* = 3,094)				
EAT volume (cm^3^)	75.05 ± 29.86	95.43 ± 43.94	75.95 ± 30.89	<0.001
PARF volume (cm^3^)	21.28 ± 11.10	29.29 ± 15.41	21.64 ± 11.44	<0.001
TAT volume (cm^3^)	7.05 ± 3.87	9.05 ± 4.89	7.14 ± 3.94	<0.001
EAT volume/BSA (cm^3^/m^2^)	39.14 ± 14.20	50.50 ± 20.60	39.60 ± 14.80	<0.001
PARF volume/BSA (cm^3^/m^2^)	11.00 ± 5.20	15.50 ± 7.50	11.20 ± 5.40	<0.001
TAT volume/BSA (cm^3^/m^2^)	3.62 ± 1.81	4.73 ± 2.31	3.67 ± 1.85	<0.001
Echocardiography (*n* = 2,129)				
LV septal wall thickness (mm)	9.21 ± 1.15	9.80 ± 1.36	9.25 ± 1.17	<0.001
LV posterior wall thickness (mm)	9.19 ± 1.07	9.74 ± 1.07	9.22 ± 1.08	<0.001
LV internal diameter (mm)	46.88 ± 3.59	47.53 ± 4.37	46.92 ± 3.64	0.047
LV mass (g)	147.61 ± 32.94	164.54 ± 36.78	148.67 ± 33.44	<0.001
LVMI (g/m^2^)	77.35 ± 14.64	85.49 ± 14.84	77.86 ± 14.78	<0.001
LV hypertrophy, *n* (%)	31 (1.6%)	5 (3.8%)	36 (1.7%)	0.057
LV geometry, *n* (%)				
No hypertrophy or remodeling	1,540 (77.2%)	87 (65.4%)	1,627 (76.4%)	0.003
Concentric remodeling	420 (21.0%)	41 (30.8%)	461 (21.7%)	
Eccentric hypertrophy	17 (0.9%)	4 (3.0%)	21 (1.0%)	
Concentric hypertrophy	19 (1.0%)	1 (0.8%)	20 (0.9%)	

BSA, body surface area; EAT, epicardial adipose tissue; LV, left ventricle; LVMI, left ventricular mass index; PARF, peri-aortic root fat; TAT, thoracic peri-aortic adipose tissue.

Data are presented as mean ± SD unless otherwise specified.

### 3.5 Differences of intrathoracic VAT accumulation between participants with and without HF development

The HF group had significantly higher baseline volumes of EAT (95.43 ± 43.94 cm^3^ vs. 75.05 ± 29.86 cm^3^), PARF (29.29 ± 15.41 cm^3^ vs. 21.28 ± 11.10 cm^3^), and TAT (9.05 ± 4.89 cm^3^ vs. 7.05 ± 3.87 cm^3^) compared to the non-HF group, even after indexation of these volumes to BSA (all *p* < 0.001). (Table 2).

**Figure 4:**
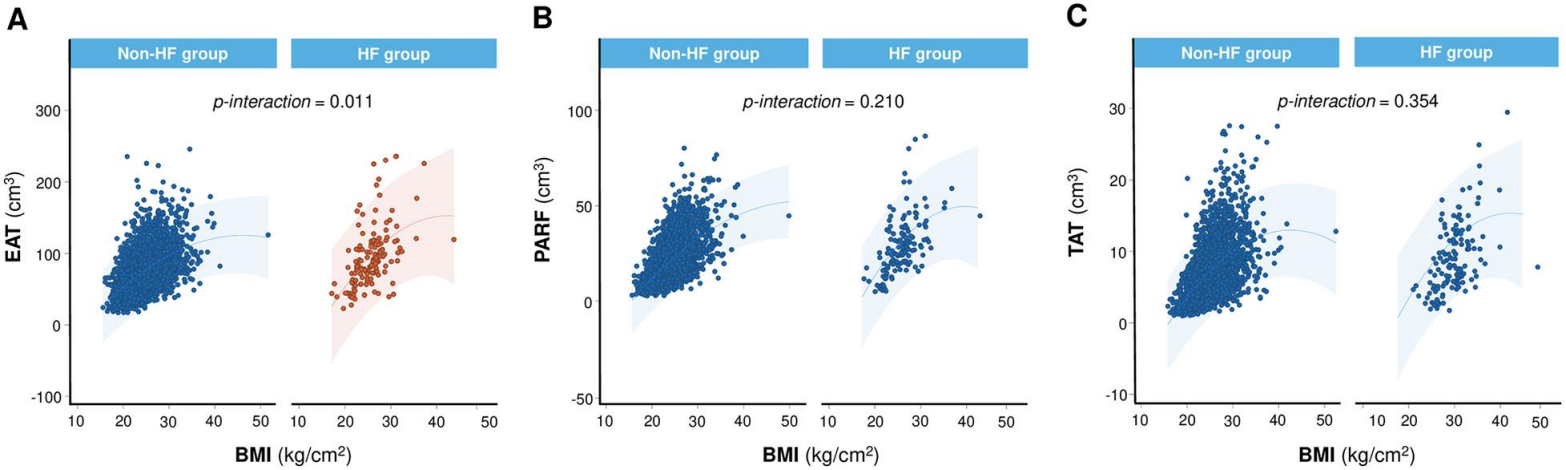
Association between BMI and intrathoracic visceral adiposity in HF and Non-HF groups. Caption: Scatter plots showing the association between body mass index (BMI) and **(A)** epicardial adipose tissue (EAT), **(B)** peri-aortic root fat (PARF), and **(C)** thoracic peri-aortic adipose tissue (TAT) in the HF group vs. non-HF group. Fitted regression lines with 95% confidence intervals (shaded areas) illustrate trends in adiposity volume across BMI levels.

As BMI increased, EAT volume rose more markedly in the HF group than in the non-HF group (*p*-interaction = 0.011) (Figure 4A). In contrast, increases in PARF and TAT volumes with rising BMI did not differ significantly between groups (*p*-interaction = 0.210 and 0.354, respectively) (Figures 4B,C), indicating more uniform fat accumulation regardless of HF development. These findings suggest that increasing EAT volume is more strongly associated with HF and may play a more prominent role in its pathogenesis.

### 3.6 Association of intrathoracic VAT with incident HF

In multivariable Cox regression analyses stratified by different anthropometric indicators of obesity (BMI and waist circumference [WC]), higher volumes of EAT, PARF, and TAT were associated with an increased risk of incident HF, with optimal cutoffs estimated to be 71.37 cm^3^, 23.50 cm^3^, and 9.59 cm^3^, respectively. Stepwise adjustment of covariates is presented in Table 3.

**Table 3:** Multivariable Cox regression models examining the association between intrathoracic visceral adiposity and incident heart failure .

	EAT (per + 1 *Z* score)		PARF (per + 1 *Z* score)		TAT (per + 1 *Z* score)	
	Hazard ratio (95% CI)	*p*-value	Hazard ratio (95% CI)	*p*-value	Hazard ratio (95% CI)	*p*-value
BMI-based models						
Unadjusted model	1.76 (1.54–2.01)	<0.001	1.72 (1.52–1.95)	< 0.001	1.62 (1.43–1.84)	<0.001
Model 1	1.43 (1.25–1.64)	<0.001	1.49 (1.30–1.70)	<0.001	1.33 (1.16–1.53)	<0.001
Model 2	1.31 (1.16–1.54)	0.001	1.38 (1.17–1.64)	<0.001	1.25 (1.04–1.50)	0.020
Model 3	1.23 (1.04–1.46)	0.013	1.31 (1.09–1.57)	0.003	1.13 (0.93–1.37)	0.210
Model 4	1.20 (1.01–1.43)	0.034	1.28 (1.07–1.54)	0.008	1.10 (0.90–1.35)	0.340
WC-based models						
Unadjusted model	1.76 (1.54–2.01)	<0.001	1.72 (1.52–1.95)	<0.001	1.62 (1.43–1.84)	<0.001
Model 1	1.43 (1.25–1.64)	<0.001	1.49 (1.30–1.70)	<0.001	1.33 (1.16–1.53)	<0.001
Model 2	1.41 (1.22–1.63)	<0.001	1.45 (1.25–1.68)	<0.001	1.38 (1.17–1.63)	<0.001
Model 3	1.34 (1.15–1.56)	<0.001	1.39 (1.19–1.62)	<0.001	1.26 (1.06–1.49)	0.009
Model 4	1.28 (1.09–1.50)	0.002	1.37 (1.17–1.60)	<0.001	1.22 (1.02–1.46)	0.028

For BMI-based models, model 1 was adjusted for age; model 2 was adjusted for age, sex, and BMI; Model 3 was adjusted for age, sex, BMI, and covariates including hypertension, cardiovascular diseases, diabetes mellitus, hyperlipidemia, estimated glomerular filtration rate (eGFR), left ventricular mass, and smoking; Model 4 was adjusted for age, sex, BMI, and covariates including hypertension, cardiovascular diseases, diabetes mellitus, hyperlipidemia, eGFR, LVEDV, and smoking.

For WC-based models, model 1 was adjusted for age; model 2 was adjusted for age, sex, and WC; Model 3 was adjusted for age, sex, WC, and covariates including hypertension, cardiovascular diseases, diabetes mellitus, hyperlipidemia, eGFR, left ventricular mass, and smoking; Model 4 was adjusted for age, sex, WC, and covariates including hypertension, cardiovascular diseases, diabetes mellitus, hyperlipidemia, eGFR, LVEDV, and smoking.

BMI, body mass index; EAT, epicardial adipose tissue; LVEDV, left ventricular end-diastolic volume; PARF, peri-aortic root fat; TAT, thoracic peri-aortic adipose tissue; WC, waist circumference.

In unadjusted models based on BMI, the hazard ratios (HRs) per 1-unit increase in the *Z* score of EAT, PARF, and TAT volume were 1.76 (95% CI: 1.54–2.01; *p* < 0.001), 1.72 (95% CI: 1.52–1.95; *p* < 0.001), and 1.62 (95% CI: 1.43–1.84; *p* < 0.001), respectively. These associations remained significant after adjustment for age (Model 1) and further for sex and BMI (Model 2), though attenuated. In the fully-adjusted model (Model 3), which additionally accounted for traditional cardiovascular risk factors—including hypertension, CVD, DM, hyperlipidemia, and smoking—as well as estimated glomerular filtration rate (eGFR) and LV mass, only EAT (HR: 1.23; 95% CI: 1.04–1.46; *p* = 0.013) and PARF (HR: 1.31; 95% CI: 1.09–1.57; *p* = 0.003) remained independently associated with HF risk, while the association for TAT was no longer significant (HR: 1.13; 95% CI: 0.93–1.37; *p* = 0.210). In a sensitivity analysis (Model 4), LV mass was replaced by left ventricular end-diastolic volume (LVEDV) to account for cardiac size and potential pericardial cavity expansion that may influence EAT volume, with all other covariates unchanged from Model 3. The findings were comparable to those of Model 3, with EAT (HR: 1.20; 95% CI: 1.01–1.43; *p* = 0.034) and PARF (HR: 1.28; 95% CI: 1.07–1.54; *p* = 0.008) remaining significantly associated with HF risk, whereas TAT remained non-significant (HR: 1.10; 95% CI: 0.90–1.35; *p* = 0.340).

In contrast, in WC-based models, EAT, PARF, and TAT all remained significantly associated with incident HF across all levels of adjustment. In the fully adjusted model (Model 3), the HRs per 1-unit increase in the *Z* score of EAT, PARF, and TAT volume were 1.34 (95% CI: 1.15–1.56; *p* < 0.001), 1.39 (95% CI: 1.19–1.62; *p* < 0.001), and 1.26 (95% CI: 1.06–1.49; *p* = 0.009), respectively. Sensitivity analyses replacing LV mass with LVEDV (Model 4) yielded consistent results.

**Figure 5:**
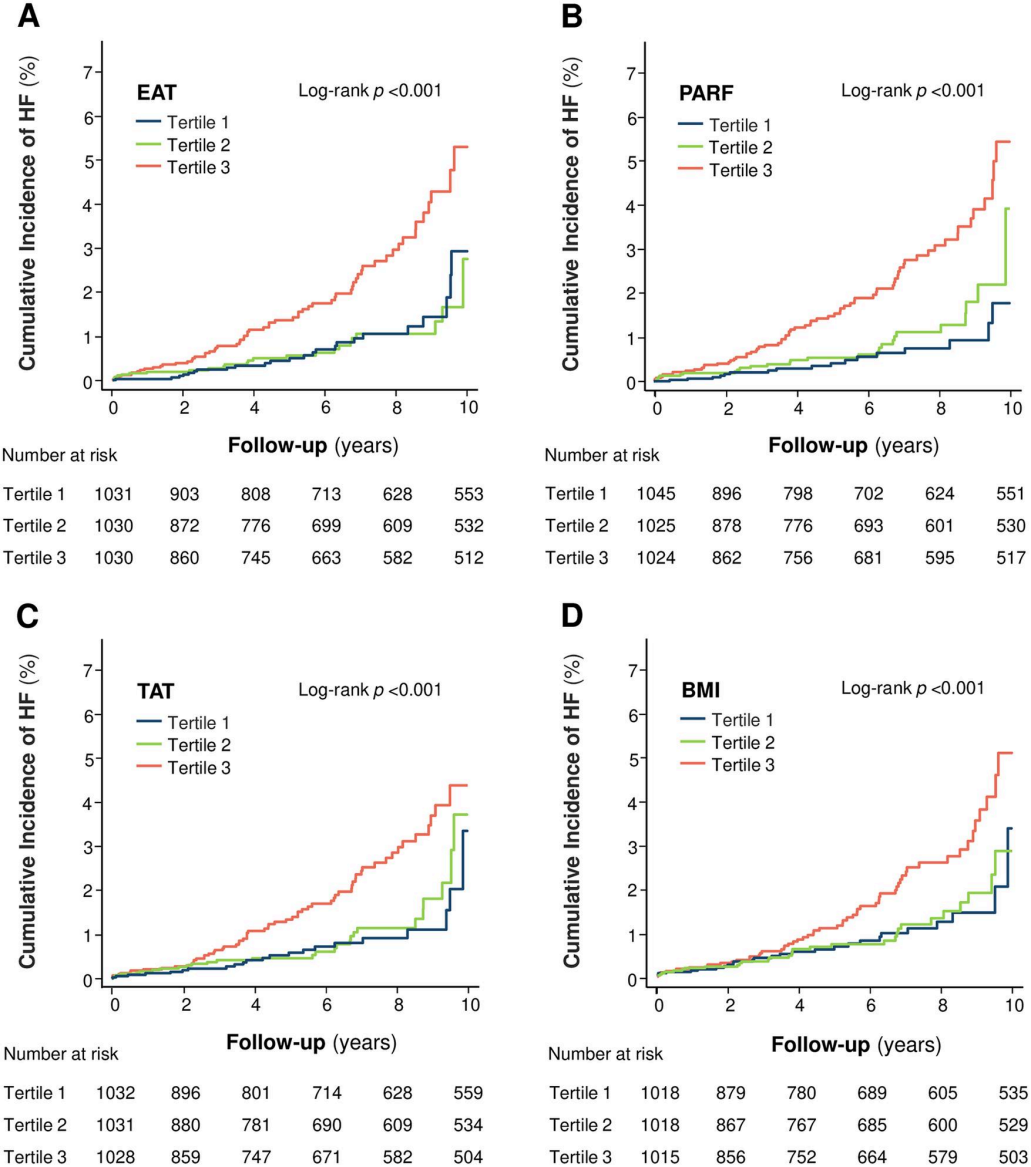
Cumulative incidence of HF by intrathoracic visceral adiposity and BMI tertiles. Caption: Kaplan–Meier Curves for cumulative incidence of heart failure is shown by tertiles of **(A)** epicardial adipose tissue (EAT), **(B)** peri-aortic root fat (PARF), **(C)** thoracic peri-aortic adipose tissue (TAT), and **(D)** body mass index (BMI). Number at risk is displayed below each plot.

Harrell's concordance indices demonstrated moderate predictive discrimination of these depots (EAT: 0.654; PARF: 0.660; TAT: 0.622). Kaplan–Meier analyses showed significant differences in cumulative HF incidence across tertiles of BMI, EAT, PARF, and TAT, with higher tertiles showing increased cumulative HF incidence (log-rank *p* < 0.001 for all; chi-square: 18.69, 30.97, 43.77, and 28.81, respectively) (Figure 5).

## Discussion

In this large cohort of asymptomatic individuals evaluated using cardiac CT, we found that elevated volumes of EAT, PARF, and TAT, as well as higher BMI, were associated with an increased risk of HF, primarily HFpEF. Among these, EAT and PARF demonstrated independent associations with incident HF. Notably, EAT showed the strongest correlations with LVMI and HF development.

Our findings align with recent large cohort studies, such as the MESA reported by Kenchaiah et al., which demonstrated that increasing CT-quantified pericardial fat volume was associated with a linear rise in the risk of incident HF, particularly HFpEF, without significant racial differences (9). Their elegant work highlighted the relevance of intrathoracic VAT in HF development; however, pericardial fat was assessed as a single composite depot combining epicardial (EAT) and paracardial (located external to the parietal pericardium) fat compartments. Distinguishing these compartments may be important, as EAT is a well-established cardiovascular risk factor (10, 16), whereas the pathophysiological significance of paracardial fat remains less clearly defined. Similarly, the Rotterdam Study identified CT-quantified EAT volume as a predictor of incident HF and subclinical cardiac dysfunction in the general Dutch population, although it focused exclusively on EAT and did not differentiate between HF phenotypes (8). Our study extends prior work by providing a more granular, anatomically detailed assessment through separate quantification of EAT, PARF, and TAT. Notably, EAT and PARF demonstrated independent associations with incident HF after adjustment for anthropometric measures (BMI and WC), traditional cardiovascular risk factors, and cardiac structural parameters (LV mass and LVEDV). In contrast, the association for TAT was sensitive to the anthropometric measure used and reached significance only in WC-based models across different levels of adjustment. Taken together, these findings suggest a potential role for EAT and PARF as imaging markers for identifying individuals at increased risk of HF.

Several plausible mechanisms may underlie these associations. First, EAT is a metabolically active VAT depot with endocrine and paracrine functions that regulate cardiovascular homeostasis (28). In obesity, the expanded and dysfunctional EAT triggers the secretion of proinflammatory adipokines that contribute to coronary atherosclerosis, myocardial fibrosis, and atrial fibrillation (AF) (29–33). Second, direct fatty infiltration of the left atrium by EAT may result in conduction abnormalities, further increasing the risk of AF (23, 34, 35). Lastly, excessive fat accumulation within the pericardium can exert a physical constraint on the heart, impairing diastolic function and elevating left-sided filling pressures through ventricular interdependence (7, 28). In contrast, PARF and TAT do not directly contact the myocardium and are therefore more likely to influence HF risk through systemic effects. Supporting this notion, our previous work demonstrated that greater PARF volumes are associated with metabolic syndrome, as well as adverse vascular remodeling and plaque formation in carotid arteries, independent of traditional cardiometabolic risk factors (21). Similarly, TAT has been linked to systemic inflammation, insulin resistance, and metabolic syndrome, and has also been associated with large vessel stiffness (20, 22, 36–38). Whilst both depots may act through systemic pathways, PARF demonstrated a stronger independent link to HF development than TAT in our study. Notably, our findings demonstrated a positive correlation between intrathoracic VAT and LVMI, particularly for EAT, consistent with prior studies reporting similar relationships with EAT thickness or volume (39–42). This association may be partly related to the mechanisms described above and the elevated hemodynamic load observed in obesity (43), which is associated with LV overload and hypertrophy and may have implications for HF risk. However, cardiac adaptations to increased adiposity are complex, as reflected by the observed non-linear relationship between BMI and LVMI, which diverges between non-obese and obese BMI ranges, suggesting that these associations are not uniform across levels of adiposity; the underlying mechanisms are beyond the scope of this study and warrant further investigation.

HFpEF is known to exhibit ethnic and gender differences. Asian patients with HFpEF tend to present at a relatively younger age and with lower BMI compared to their Western counterparts (44–46). In our study, the mean age (65.2 ± 11.4 years) and BMI (26.87 ± 4.51) of participants at the time of HF onset were comparable to data from large Asian cohorts (45). Furthermore, HFpEF occurs nearly twice as often in women as in men (47). Beyond hormonal influences, evidence from MESA suggests greater female susceptibility to the negative effects of intrathoracic VAT, with a significantly stronger association between pericardial fat and HFpEF risk among women compared with men (HR per 1-SD increase: 1.44 vs. 1.13; *p*-interaction = 0.01) (9, 48). In line with this, our study found that despite having lower volumes of EAT, PARF, and TAT, and a less pronounced accumulation of these depots with rising BMI, women nevertheless exhibited a greater cumulative risk of HF compared to men (5.61% vs. 4.11%). This heightened susceptibility is believed to reflect sex-specific differences in fat distribution. For instance, Zhu et al. found that in patients with AF, women demonstrated a higher periatrial-to-total EAT ratio despite having lower total EAT volumes than men (49). Furthermore, in patients with HFpEF, women exhibited significantly higher intramyocardial fat compared to men based on magnetic resonance imaging (MRI) quantification techniques (50). Menopausal hormonal changes may further exacerbate this disparity, as declining estrogen levels have been shown to promote VAT accumulation (48). Supporting this, Kim et al. reported that EAT thickness measured by echocardiography was significantly greater in women than in men among individuals aged 60 years or older, despite no sex differences below this age threshold (51). Likewise, in patients with type 2 DM, postmenopausal women had higher CT-quantified EAT volumes compared to premenopausal women, and EAT was independently associated with diastolic dysfunction in postmenopausal women and men, but not in premenopausal women (52). Collectively, these findings raise the possibility of an accelerated accumulation of EAT during the menopausal transition, potentially contributing to women's increased vulnerability to the adverse cardiac effects of intrathoracic VAT.

The widespread adoption of cardiac CT for coronary artery assessment offers a valuable opportunity to obtain additional prognostic information by quantifying distinct VAT depots, with no extra radiation exposure since they are captured within the same scan range. Incorporating these CT–derived imaging biomarkers, particularly EAT and PARF, into clinical risk assessment may enhance early identification of individuals at risk for HFpEF. This approach is especially pertinent with the advent of sodium–glucose co-transporter 2 (SGLT2) inhibitors and glucagon-like peptide-1 (GLP-1) receptor agonists—novel pharmacologic therapies for HFpEF—that have also been shown to reduce EAT volume in individuals with obesity or type 2 DM (53–56). Further investigation is needed to determine whether these agents can also mitigate long-term HF risk in asymptomatic individuals with elevated intrathoracic VAT.

## 5 Limitations

This study has several limitations. As a single-center retrospective observational study, it is subject to selection bias and precludes causal inference. Second, the proportion of HFpEF in our cohort (89.9%) was higher than previously reported (19%–55%) (57). This discrepancy may partly reflect survivor bias, whereby higher early mortality among patients with HFrEF may lead to underestimation of its incidence and a relative overrepresentation of HFpEF under the study outcome definition, potentially introducing classification bias that should be considered when interpreting the findings. In addition, the relatively small and imbalanced number of HF cases limited meaningful subgroup analyses of HFpEF and HFrEF in relation to intrathoracic VAT. Third, the study population consisted exclusively of Asian individuals, which may limit generalizability to other ethnic groups. Fourth, although women appeared more susceptible to intrathoracic VAT, the influence of menopausal status was not assessed and warrants further investigation. Fifth, the TAT quantified in this study represents only a portion of the adipose tissue surrounding the thoracic aorta, rather than the total volume along its entire length. While this approach standardizes measurement, it may not fully capture overall TAT burden. Finally, analyses of the relationships between intrathoracic VAT, BMI, and LVMI were exploratory, and the choice of indexation method may influence these findings. Future studies should consider alternative approaches to better account for differences in body and cardiac size.

## 6 Conclusion

This study highlights the prognostic significance of distinct intrathoracic VAT depots, particularly EAT and PARF, in the development of incident HF, predominantly HFpEF. These results underscore the potential utility of cardiac CT–based quantification of intrathoracic VAT as a novel tool for early HF screening and improved risk stratification, particularly among women, who appeared more susceptible to its adverse effects.

## Data Availability

The raw data supporting the conclusions of this article will be made available by the authors, without undue reservation.
