# Supplementary material for: Quantitative CT assessment of intrathoracic visceral fat depots and their association with incident heart failure in asymptomatic adults
Source: Front Cardiovasc Med. 2026 May 28;13:1820542. doi: 10.3389/fcvm.2026.1820542 (PMC13253433; doi:10.3389/fcvm.2026.1820542)
Supplement: Supplementary file 1 [file Datasheet1.docx]

Supplementary Material

#
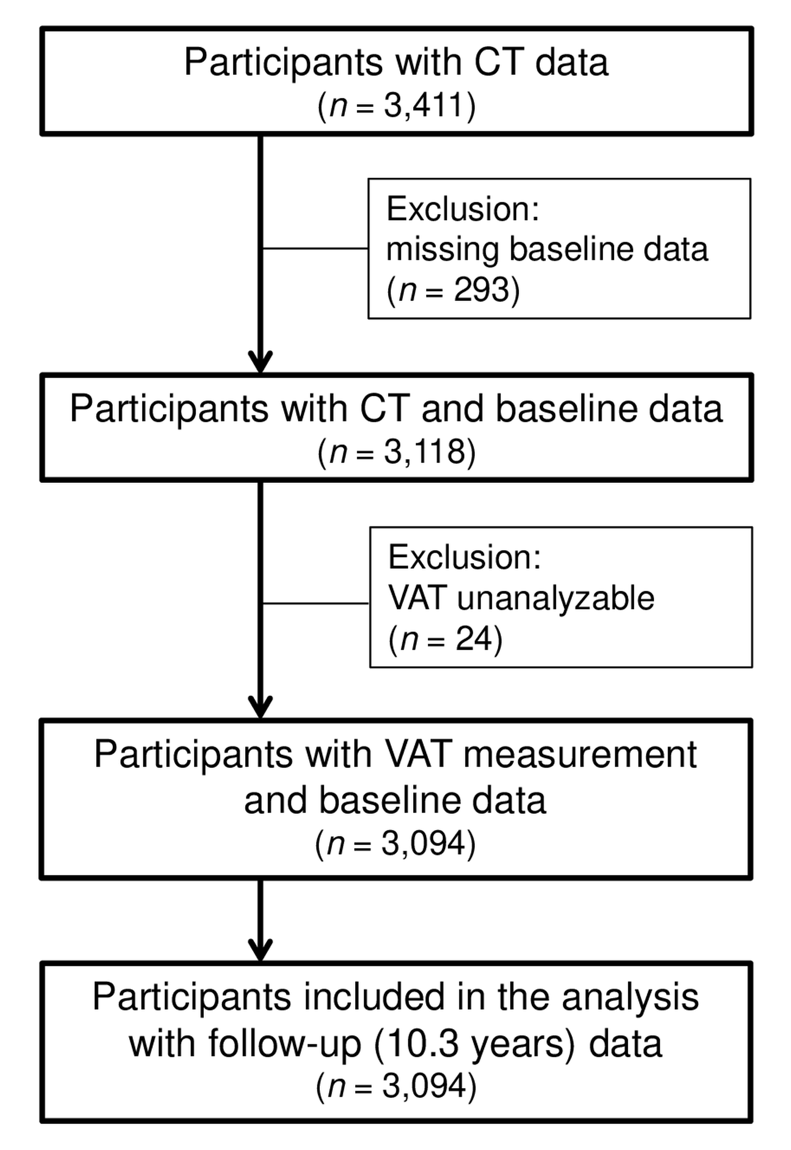
Supplementary Figure 1. Flowchart Depicting the Participant Selection Process in the Study

| **Supplementary Table 1. Subgroup Comparison of HFpEF vs HFrEF Patients Within the HF Group: Baseline Characteristics and Clinical Features at the Time of HF Onset** | | | | |
| --- | --- | --- | --- | --- |
|  | All HF (*n* = 140) | HFpEF (*n* = 107) | HFrEF (*n* = 12) | *p-*value |
| Baseline Characteristics | | | | |
| EAT volume (cm^3^) | 95.43 ± 43.94 | 97.5 ± 40.6 | 81.45 ± 54.0 | 0.23 |
| PARF volume (cm^3^) | 29.29 ± 15.41 | 30.6 ± 14.3 | 25.1 ± 1 | 0.24 |
| TAT volume (cm^3^) | 9.05 ± 4.89 | 10.1 ± 5.1 | 7.53 ± 5.0 | 0.12 |
| BMI (kg/m^2^) | 26.87 ± 4.51 | 26.2 ± 3.8 | 25.8 ± 5.1 | 0.73 |
| LVMI (g/m^2^) | 85.8 ± 15.1 | 91.9 ± 17.4 | 85.1 ± 14.8 | 0.14 |
| Clinical Features at the Time of HF Onset | | | | |
| Age (years) | 64.5 ± 11.5 | 65.1 ± 1.08 | 59.8 ± 13.2 | 0.13 |
| Sex |  |  |  |  |
| Male, *n* (%) | 92 (65.7%) | 71 (66.4%) | 10 (83.3%) | 0.23 |
| Female, *n* (%) | 48 (34.3%) | 36 (33.6%) | 2 (16.7%) |  |
| LVEF (%) | 57.8 ± 8.3 | 60.0 ± 4.8 | 37.8 ± 6.2 | <0.001 |
| SBP (mmHg) | 164.4 ± 33.6 | 128.8 ± 19.0 | 133.8 ± 21.2 | 0.39 |
| DBP (mmHg) | 85.3 ± 13.7 | 77.3 ± 10.8 | 80.9 ± 9.9 | 0.26 |
| Pulse rate (bpm) | 73.5 ± 10.3 | 72.6 ± 9.8 | 83.5 ± 12.7 | 0.001 |

Abbreviations: BMI, body mass index; DBP, diastolic blood pressure; HFpEF, heart failure with preserved ejection fraction; HFrEF, heart failure with reduced ejection fraction; LVEF, left ventricular ejection fraction; LVMI, left ventricular mass index; SBP, systolic blood pressure.
